# Dispensaries

**Published:** 1886-07

**Authors:** 


### Dispensaries.

In our last issue we called the attention of our readers to the fact that there was a law recently passed empowering the Board of Supervisors of this county to appropriate money to assist certain dispensaries in this city. At this time we added a vigorous protest against such action. At the recent meeting of the Erie County Medical Society, the subject came before the society, on a resolution offered, instructing the Committee on Legislation to oppose, before the Board of Supervisors, any appropriation for dispensary purposes. The sentiment of the society was unanimously in favor of such action. One of the members of the society offered, as a substitute, an amendment, endorsing the dispensary now drawing money from the County Treasury, but opposing any further appropriation. This was promptlyrejected; at the same time, it seemed to be the sentiment of those present that good work was being done by the gentleman interested in the Buffalo Eye and Ear Infirmary, and that proper discrimination was used in the granting of relief. It was held, however, and we think rightly, that the society, in condemning the policy, could not make any discrimination.

The committee also were instructed to present the so-called Medical Faculty Bill, which was originated by this Society, to our Senator and to one of the Assemblymen from this district, with the request that it be introduced and its passage urged in the next Legislature. This measure has a good chance of success, if properly pushed. It is to be hoped that in our Senator from this city we have a man who will see the merits and need of such a measure.

Thus the society accomplished much in a few short hours. We would suggest the Board of Censors turn their attention to the many druggists in this city who prescribe for patients over their counters. Many such can be found. The sooner they are prosecuted the better.

